# Predictors of Plasma DDT and DDE Concentrations among Women Exposed to Indoor Residual Spraying for Malaria Control in the South African Study of Women and Babies (SOWB)

**DOI:** 10.1289/ehp.1307025

**Published:** 2014-02-21

**Authors:** Kristina W. Whitworth, Riana M.S. Bornman, Janet I. Archer, Mwenda O. Kudumu, Gregory S. Travlos, Ralph E. Wilson, Matthew P. Longnecker

**Affiliations:** 1 The University of Texas School of Public Health, San Antonio Regional Campus, San Antonio, Texas, USA; 2 Department of Urology, and; 3 The University of Pretoria Centre for Sustainable Malaria Control, University of Pretoria, Pretoria, South Africa; 4 Social and Scientific Systems Inc., Durham, North Carolina, USA; 5 Cellular and Molecular Pathology Branch, and; 6 Epidemiology Branch, National Institute of Environmental Health Sciences, National Institutes of Health, Department of Health and Human Services, Research Triangle Park, North Carolina, USA

## Abstract

Background: Few studies have examined predictors of DDT (dichlorodiphenyltrichloroethane) and DDE (dichlorodiphenyldichloroethylene) levels among residents in homes sprayed with DDT for malaria control with the aim of identifying exposure-reduction strategies.

Methods: The present analysis included 381 women enrolled in the Study of Women and Babies (SOWB) during 2010–2011, from eight South African villages in the Limpopo Province, South Africa. Indoor residual spraying (IRS) occurred in half of the villages. Questionnaires regarding various demographic and medical factors were administered and blood samples were obtained. We classified the women into three exposure groups by type of residence: unsprayed village (*n* = 175), IRS village in household with a low likelihood of DDT use (non-DDT IRS household, *n* = 106), IRS village in household with a high likelihood of DDT use (DDT IRS household, *n* = 100). We used multivariable models of natural log-transformed DDT plasma levels (in micrograms per liter) and DDE (in micrograms per liter) to identify predictors for each group.

Results: Median levels of DDT and DDE among women in unsprayed villages were 0.3 [interquartile range (IQR): 0.1–0.9] and 1.7 (IQR: 0.7–5.5), respectively. Median levels of DDT and DDE among women in DDT IRS households were 2.6 (IQR: 1.1–6.6) and 8.5 (IQR: 4.7–18.0), respectively. In unsprayed villages, women with water piped to the yard, rather than a public tap, had 73% lower DDT (95% CI: –83, –57%) and 61% lower DDE (95% CI: –74, –40%) levels. In DDT IRS households, women who reported taking more than six actions to prepare their home before IRS (e.g., covering water and food) had 40% lower DDT levels (95% CI: –63, –0.3%) than women who took fewer than four actions.

Conclusion: The predictors of DDT and DDE plasma levels identified in the present study may inform interventions aimed at decreasing exposure. Among households where DDT is likely to be used for IRS, education regarding home preparations may provide an interventional target.

Citation: Whitworth KW, Bornman RM, Archer JI, Kudumu MO, Travlos GS, Wilson RE, Longnecker MP. 2014. Predictors of plasma DDT and DDE concentrations among women exposed to indoor residual spraying for malaria control in the South African Study of Women and Babies (SOWB). Environ Health Perspect 122:545–552; http://dx.doi.org/10.1289/ehp.1307025

## Introduction

Despite concerns regarding the health effects of exposure to DDT (dichlorodiphenyltrichloroethane), it continues to be used for vector control in some developing countries (Bouwman 2004). In 2001, > 100 nations signed the Stockholm Convention on Persistent Organic Pollutants, aiming to significantly reduce or completely eliminate the use of 12 persistent organic pollutants, including DDT (Stockholm Convention 2008). The Stockholm Convention included a provision for DDT use in malaria control and, as such, it continues to be used in some malaria-endemic countries, including South Africa. DDT and its primary degradation product and metabolite, DDE (dichlorodiphenyldichloroethylene), are lipophilic compounds that are persistent in the environment and have the ability to bioaccumulate. DDT is classified as a “possible carcinogen” by the International Agency for Research on Cancer (1991). The epidemiologic literature also suggests an association between DDT exposure and a variety of health effects, including increased risk of diabetes, impaired reproduction, and adverse effects on childhood neurodevelopment (Eskenazi et al. 2009).

Recent studies suggest elevated plasma levels of DDT and DDE among workers who apply DDT during indoor residual spraying (IRS), as well as among residents in areas where IRS takes place, compared with levels from the general population (Channa et al. 2012; de Jager et al. 2009; Ritter et al. 2011; Wassie et al. 2012). Despite evidence of potential harmful human health effects of DDT exposure, there remains a gap in knowledge regarding determinants and strategies for reduction of exposure, particularly among nonoccupationally exposed individuals (i.e., residents of IRS-treated homes). Few studies have examined determinants of body burden among this population, and those that have suggest that inhalation and food consumption are relevant routes of exposure (Gyalpo et al. 2012; Ritter et al. 2011). Although the majority of South Africa is considered a low-risk malaria area, malaria continues to be endemic in the Limpopo Province and IRS remains a mainstay of vector control (Moonasar et al. 2012). Although both pyrethroids and DDT are used in IRS, mosquito species resistant to pyrethroids have emerged; therefore, DDT use has been stepped up since 2000 and, in some cases, is considered the best option for vector control (Blumberg and Frean 2007; Gerritsen et al. 2008; Hargreaves et al. 2000).

The goal of the present study was to quantify plasma DDT and DDE concentrations among reproductive-aged women living in the Vhembe District of the Limpopo Province and to examine the determinants of plasma DDT and DDE levels, considering the contributions from demographic, reproductive, dietary, housing, and IRS-related factors.

## Methods

*Study area and sampling procedure*. We analyzed data from the Study of Women and Babies (SOWB), a study designed to examine DDT exposure in relation to clinically recognized pregnancy loss. During 2010–2011, 442 women were enrolled from eight villages in the Thulamela Municipality of the Vhembe District of the Limpopo Province, South Africa. Study villages were intentionally selected such that IRS was routinely conducted in half and no IRS was conducted in the other half. To be eligible for the SOWB, women were 20–30 years of age, were not currently using hormonal contraception or an intrauterine device, had regular menstrual periods (unless currently breastfeeding), had a negative spot pregnancy test, had no previous problems becoming pregnant, had no medical or other condition that would prevent pregnancy, and were planning to reside in the same village throughout participation in the study.

Study participants were identified and recruited in several ways. Tshivenda-speaking study staff, hired locally from each study village and trained in recruitment methods, attended monthly village meetings and visited local stores, schools, nurseries, and clinics to publicize the study and distribute recruiting materials, including relevant contact information. Additional recruiting was done by word of mouth.

The present study was approved by institutional review boards of the University of Pretoria, South Africa, and the National Institute of Environmental Health Sciences (NIEHS), National Institutes of Health.

*Data collection*. Recruiting occurred by trained native-speaking study staff at local clinics, where women were administered a screening questionnaire to determine their eligibility and they provided informed consent. Consenting, eligible women were then administered a baseline questionnaire regarding demographic (age, marital status) and socioeconomic status (income and education), consumption of local foods, type of housing (type of materials used for walls and floors) and water supply (public or private), information related to IRS (whether spills occurred, whether pesticide came into contact with household items, whether any specific actions were taken before IRS occurred), and reproductive history (number of previous pregnancies and breastfeeding history). The questionnaire was pretested for cognitive assessment and cultural considerations, back and forward translated into Tshivenda, and certified by professional translators. Based on advice received from the University of Pretoria ethics committee, women were reimbursed 180 rand (approximately 27.50 US$) for completing the baseline visit. However, they were not informed of the specific reimbursement amount beforehand; they were only told that they would “be compensated for reasonable costs incurred to participate, such as transportation.”

During the baseline assessment at the local clinics, study staff also performed a physical exam using a mounted stadiometer to measure height, digital scales to measure weight, and nonstretchable measuring tapes to measure waist circumference. Blood samples were collected at this time by a phlebotomist hired specifically for this study. Blood samples were collected in the clinic in a separate phlebotomy room, and kept in a cooler with cold packs during clinic hours. On the same day, samples were transported approximately 5 miles to the field office for processing. During processing, staff handled the blood samples one specimen at a time to ensure labels were correctly applied and to avoid intersample contamination. Plasma blood collection tubes for DDT/DDE analysis were processed in a designated secure specimen processing and storage area in the field office. Plasma samples were immediately frozen in a –20°C freezer, which was equipped with a back-up generator and kept in a locked specimen storage section of the field office. Frozen samples were transported to the University of Pretoria on a weekly basis, using a specialized freezer powered by the vehicle battery. At the University of Pretoria, samples were stored in –20°C monitored freezers, where they remained frozen until shipment for analyses.

Among the 442 women initially enrolled, 15 were later found to be ineligible due to age (*n* = 3) or residence outside of the study villages (*n* = 12). In addition, a blood specimen could not be obtained from 1 otherwise eligible woman, leaving 426 women in the present analysis.

*Analytical method.* DDT and DDE concentrations were measured by the Institute national de sante publique du Quebec (INSPQ; Sainte-Foy, Quebec, Canada) using gas chromatography–mass spectrometry (GC-MS). INSPQ routinely participates in an international laboratory comparison program coordinated by the University of Erlangen-Nuremberg (Erlangen and Nuremberg, Germany), and their results for DDT and DDE are consistently in good agreement with those from the > 10 other participating laboratories. Specifically, 2 mL of plasma samples were enriched with carbon-13–labeled internal standards (*p,p*´-DDE-^13^C_12_ and *p,p*´-DDT-^13^C_12_) and proteins were denatured with reagent alcohol. Organochlorinated compounds were extracted with hexane from the aqueous matrix using a liquid–liquid extraction. The extracts were evaporated to dryness before they were dissolved in 0.5 mL hexane. These extracts were cleaned up on activated florisil columns and eluted with a mixture of dichloromethane:hexane (9 mL; 25:75 vol:vol) before analysis by GC-MS. The solvent was evaporated, the residue was dissolved in 50 μL hexane, and then the extract was analyzed for *p,p*´-DDE and *p,p*´-DDT on an Agilent 6890 Network GC equipped with a 7683B Series automatic injector and a 5975 MS. The GC was fitted with a 15-m DB-XLB column (0.25-mm i.d., 0.10-μm film thickness) to the MS (all from Agilent Technologies, Santa Clara, CA, USA). The run time for the analysis was 16.3 min. The carrier gas was helium and all injections were 1 μL in pulsed splitless mode. The MS was operated in selected ion monitoring, using negative chemical ionization with methane (99.97%) as the reagent gas. Analyte concentrations were evaluated by considering the percent recovery of labeled internal standards. The limit of quantification (LOQ) for both DDT and DDE was 0.02 μg/L. Values below the LOQ were assigned a value of one-half the LOQ. Specimens were analyzed in 14 batches; all batches except one contained an aliquot from a single quality assurance/quality control (QA/QC) specimen, consisting of pooled material from 13 of the consented ineligible subjects who provided blood samples. Laboratory technicians were blinded to the identity of the QA/QC samples. The mean DDT level among the 13 QA/QC samples was 1.9 μg/L, and the between-batch coefficient of variation was 5.0%. The mean DDE level among the 13 QA/QC samples was 9.4 μg/L and the between-batch coefficient of variation was 7.8%. Because DDT and DDE concentrations were skewed with a long tail to the right, we used natural log-transformed values for all analyses. We used plasma samples to measure triglycerides (TG) and total cholesterol (TC) levels (both in milligrams per deciliter) at the NIEHS, using an AU400e Clinical Chemistry Analyzer (Olympus, Center Valley, PA, USA) and reagents from Beckman Coulter (Brea, CA, USA). Total lipids (TL; milligrams per deciliter) were estimated as TL = 1.3 × (TG + TC) + 90 mg/dL (Rylander et al. 2006). All analyses of DDT and DDE levels were adjusted for TL because lipids can affect the measured concentration.

*Data analysis*. Because IRS spraying in South Africa is conducted using either pyrethroids or DDT [formulated according to specifications of the World Health Organization (WHO 2009)] and no spray records for individual households were available, it was necessary to identify two distinct groups among women in IRS villages: those in households either more or less likely to have received IRS with DDT (as opposed to pyrethroids). Although several factors may contribute to the choice of selecting one pesticide over the other [e.g., presence of resistant mosquito species, cost, availability, efficacy (WHO 2006)], housing characteristics are important considerations. Whereas DDT is generally used in traditional houses with daubed walls, pyrethroids are favored in western-style houses with painted or plastered surfaces (Biscoe et al. 2005). Reconstructing whether a particular household in an IRS village would have been treated with DDT was not straightforward. Although spray records were not kept for individual homes, we did have housing data collected via a questionnaire completed at baseline and we used a statistical approach to distinguish between participants in households that were either more or less likely to have been sprayed with DDT. We identified the housing characteristics that best predicted DDT levels, using stepwise linear regression (see Supplemental Material, Table S1). We then conducted a factor analysis using PROC FACTOR in SAS (version 9.3; SAS Institute Inc., Cary, NC, USA), using identified housing characteristics (i.e., living in a traditional compound, having painted walls, having daubed walls), and water source to create a single factor (see Supplemental Material, Table S2), which was subsequently dichotomized at the median to discriminate between subjects living in a house that was more or less likely to have been sprayed with DDT. This resulted in the creation of three analytical groups: *a*) women in villages where IRS did not occur (unsprayed villages), *b*) women in IRS villages in households with a low likelihood of DDT use (non-DDT IRS households), and *c*) women in IRS villages in households with a high likelihood of DDT use (DDT IRS households).

We began by first examining relationships between potential determinants and DDT and DDE concentrations using bivariate regression analyses. The purpose of these analyses was to explore the unadjusted relation between potential determinants and DDT and DDE plasma levels. Variables were categorized either based on percentiles (natural cut points of the distribution) or as reported by the women. The following variables were examined for all three groups: age (20–22, 23–25, 26–28, > 28 years), marital status (not married/cohabitating vs. married/cohabitating), monthly family income (< 1,250, 1,250–1,999, 2,000–3,000, > 3,000 rand), education (≤ 11, 12, > 12 years of schooling), body mass index (BMI; < 21.6, 21.6–24.7, 24.8–28.3, ≥ 28.4 kg/m^2^), age at menarche (< 14, 14, 15, > 15 years), parity (nulliparous, 1, > 1), total months of breastfeeding, water source (public tap vs. piped to yard/home), farm work (yes/no), occupational insecticide use (yes/no), and livestock ownership [yes/no (primarily chickens, cattle, and goats)]. We also had data regarding self-reported frequency of consumption of > 20 foods, including grains, meats, vegetables, fruits, and dairy. We examined the foods most likely associated with DDT levels (meats, chicken, fish, milk, cheese, butter, and eggs). For each food item, the self-reported proportion that was grown or raised by the participant or locally in the village was also collected; < 10% of the women reported consuming any of the previous foods from local sources. In addition, among the two groups of women in IRS villages, we examined the following variables related to IRS: number of actions taken before the house was sprayed [11 specific actions were queried: covering food/water, taking food/water out of the house, taking furniture out of the house, moving everything to the middle of the house, closing windows/doors, closing cupboards, covering furniture, covering plates/cups/utensils, removing everything from walls, packing/covering clothing, having everyone go outside (this variable was categorized by tertiles: < 4, 4–6, > 6 actions)], number of household areas/items touched by the spray (categorized by tertiles: < 3, 3–4, > 4), pesticide spills in or around the home (as reported by the women: none, a little, a lot), whether pesticide touched the covering over food (women were asked “How much was food that was covered touched by the spray?”), and whether pesticide touched open food (women were asked “How much was open food touched by the spray?”). In this population, total months of breastfeeding was strongly correlated with parity (*r* = 0.77, *p* < 0.0001); however, because breastfeeding did not add additional information in models that included parity, it was not considered further. One woman from the unsprayed villages was missing diet information. Among participants from IRS villages, 17 women did not have complete housing information, 26 were missing IRS-related variables (16 in non-DDT IRS households, and 10 in DDT IRS households); and one woman from a non-DDT IRS household was also missing diet information. All subsequent analyses were conducted separately for the following three groups: women in unsprayed villages (*n* = 175), women in non-DDT IRS households (*n* = 106), and women in DDT IRS households (*n* = 100).

Multivariable linear regression models of DDT and DDE were fitted separately for each of the three groups, using a forward stepwise selection process. All variables previously mentioned were considered in multivariable analyses of each exposure group, with the exception of breastfeeding duration for reasons previously mentioned. Further, IRS-related variables were only considered in multivariable modeling of the two IRS village groups. We relied on the Akaike information criterion (AIC) to determine inclusion in or exclusion from the model. The final model was the one that resulted in an optimized (minimum) AIC. We also calculated each selected covariate’s contribution to the adjusted *R*^2^ (adj. *R*^2^) to assess its relative contribution to the fit of the final model. Total lipids was forced into each model before running the forward stepwise selection process.

To assess the impact of potentially influential points, the final data and models selected were reanalyzed using PROC ROBUSTREG in SAS (version 9.3; SAS Institute Inc.), which produces stable estimates in the presence of outliers and leverage points (Chen 2002). We also identified, for each of the three groups, observations with leverage values > 2(*k* + 1)/*n*, where *k* represents the number of parameters in the model and *n* is the number of observations (Hoaglin and Welsch 1978). Once identified, these potentially influential observations were excluded, and the forward stepwise selection process for each group was repeated.

## Results

Of the 426 participants, 4 had values of DDT below the LOQ and none had values of DDE below the LOQ. The characteristics of the women in the three village groups are presented in Table 1. A gradient in the median plasma levels of both DDT and DDE across the three groups was present (Table 2). Women in unsprayed villages had the lowest median levels of DDT [0.3 μg/L; interquartile range (IQR): 0.1–0.9] and DDE (1.7 μg/L; IQR: 0.7–5.5); women in DDT IRS households had the highest median levels of DDT (2.6 μg/L; IQR: 1.1–6.6) and DDE (8.5 μg/L; IQR: 4.7–18.0). Median levels of both DDT and DDE for each of the three village groups were statistically significantly different (at α < 0.05) from one another, as assessed using Tukey’s test for pair-wise differences.

**Table 1:** Characteristics [n (%)] of South African women 20–30 years of age, 2010–2011, by exposure group.

Characteristic	Unsprayed villages (*n *= 175)	Non-DDT IRS households (*n *= 106)	DDT IRS households (*n *= 100)
Age (years)
20–22	65 (37.1)	36 (34.0)	35 (35.0)
23–25	52 (29.7)	31 (29.2)	33 (33.0)
26–28	41 (23.4)	21 (19.8)	23 (23.0)
29–30	17 (9.7)	18 (17)	9 (9.0)
Married/cohabitating
No	107 (61.1)	64 (60.4)	70 (70.0)
Yes	68 (38.9)	42 (39.6)	30 (30.0)
Family income (rand)
< 1,250	38 (21.7)	33 (31.1)	22 (22.0)
1,250–1,999	41 (23.4)	29 (27.4)	28 (28.0)
2,000–3,000	42 (24.0)	25 (23.6)	29 (29.0)
> 3,000	54 (30.9)	19 (17.9)	21 (21.0)
Education (years)
≤ 11	86 (49.1)	60 (56.6)	58 (58.0)
12	53 (30.2)	35 (33.0)	30 (30.0)
> 12	36 (20.6)	11 (10.4)	12 (12.0)
BMI (kg/m^2^)
< 21.6	47 (26.9)	21 (19.8)	24 (24.0)
21.6–24.7	45 (25.7)	29 (27.4)	25 (25.0)
24.8–28.3	41 (23.4)	31 (29.3)	25 (25.0)
≥ 28.4	42 (24.0)	25 (23.6)	26 (26.0)
Age at menarche (years)
13	46 (26.3)	24 (22.6)	22 (22.0)
14	42 (24.0)	25 (23.6)	21 (21.0)
15	47 (26.9)	36 (34.0)	29 (29.0)
> 15	40 (22.9)	21 (19.8)	28 (28.0)
Parity
Nulliparous	40 (22.9)	21 (19.8)	12 (12.0)
1	85 (48.6)	50 (47.2)	55 (55.0)
> 1	50 (28.6)	35 (33.0)	33 (33.0)
Total breastfeeding (months)
0	40 (22.9)	22 (20.8)	14 (14.0)
1–18	56 (32.0)	35 (33.0)	35 (35.0)
19–30	39 (22.3)	23 (21.7)	24 (24.0)
> 30	40 (22.9)	26 (24.5)	27 (27.0)
Water source
Public tap	51 (29.1)	41 (38.7)	58 (58.0)
Piped to yard/home	124 (70.9)	65 (61.3)	42 (42.0)
Ever do farmwork
No	120 (68.6)	75 (70.8)	57 (57.0)
Yes	55 (31.4)	32 (29.3)	43 (43.0)
Occupational insecticide use
No	148 (84.6)	88 (83.0)	81 (81.0)
Yes	27 (15.4)	18 (17.0)	19 (19.0)
Owns livestock
No	139 (79.4)	86 (81.1)	65 (65.0)
Yes	36 (20.6)	20 (18.9)	35 (35.0)
Meat consumption
< 1 time/month	93 (53.1)	57 (53.8)	44 (44.0)
1–4 times/month	56 (32.0)	33 (31.1)	39 (39.0)
> 4 times/month	26 (14.9)	16 (15.1)	17 (17.0)
Chicken consumption
≤ 1 time/week	43 (24.6)	25 (23.6)	29 (29.0)
2 times/week	40 (22.9)	24 (22.6)	19 (19.0)
3 times/week	42 (24.0)	31 (29.3)	19 (19.0)
> 3 times/week	50 (28.6)	26 (24.5)	33 (33.0)
Egg consumption
< 1 time/month	57 (32.6)	24 (22.6)	33 (33.0)
1–6 times/month	67 (38.3)	45 (42.5)	39 (39.0)
> 6 times/month	51 (29.1)	37 (34.9)	28 (28.0)
Milk consumption
< 1 time/month	77 (44.0)	49 (46.2)	44 (44.0)
1–4 times/month	61 (34.9)	32 (30.2)	29 (29.0)
> 4 times/month	37 (21.1)	25 (23.6)	27 (27.0)
Butter consumption
< 1 time/month	41 (23.4)	34 (32.1)	33 (33.0)
≥ 1 time/month and < 1 time/day	67 (38.3)	36 (34.0)	30 (30.0)
≥ 1 time/day	67 (38.3)	36 (34.0)	37 (37.0)
Fish consumption
< 1 time/month	103 (58.9)	60 (56.6)	47 (47.0)
1–4 times/month	53 (30.3)	29 (27.4)	32 (32.0)
> 4 times/month	19 (10.9)	17 (16.0)	21 (21.0)
Cheese consumption
< 1 time/month	148 (84.6)	97 (91.5)	89 (89.0)
≥ 1 time/month	27 (15.4)	9 (8.5)	11 (11.0)
Pesticide spill in home after IRS
None	NA	38 (35.9)	36 (36.0)
A little	NA	42 (39.6)	43 (43.0)
A lot	NA	26 (24.5)	21 (21.0)
No. of actions taken before IRS
< 4	NA	38 (35.9)	29 (29.0)
4–6	NA	39 (36.8)	33 (33.0)
> 6	NA	29 (27.4)	38 (38.0)
No. of items touched by spray
< 3	NA	35 (33.0)	29 (29.0)
3–4	NA	35 (33.0)	40 (40.0)
> 4	NA	36 (34.0)	31 (31.0)
Any pesticide touched open foods
No	NA	94 (88.7)	86 (86.0)
Yes	NA	12 (11.3)	14 (14.0)
Any pesticide touched covering on foods
No	NA	81 (76.4)	74 (74.0)
Yes	NA	25 (23.6)	26 (26.0)
NA, not applicable.			

**Table 2:** Summary of DDT and DDE levels [median (IQR)] among South African women 20–30 years of age, 2010–2011, by exposure group.

Exposure group	DDT (μg/L)	DDE (μg/L)
Unsprayed villages (*n *= 175)	0.31 (0.11–0.86)	1.70 (0.70–5.50)
Non-DDT IRS households (*n *= 106)	1.40 (0.50–3.00)	7.95 (3.40–12.00)
DDT IRS households (*n *= 100)	2.60 (1.10–6.60)	8.50 (4.65–18.00)
The three exposure groups were statistically significantly different (*p* < 0.01) with regard to DDT and DDE levels, assessed using Tukey’s test for pair-wise differences.		

Among women from unsprayed villages, the most important predictor of DDT levels was water source (adj. *R*^2^ = 0.16) (Table 3). Women who had access to water piped directly into their yard or house had, on average, 73% lower DDT levels [95% confidence interval (CI): –83, –57%] compared with women who relied on a public tap. The remaining predictors of DDT levels among these women were butter and egg consumption, which, comparatively, made little contribution to the overall model fit (sum of adj. *R*^2^ = 0.05).

**Table 3:** Multivariable linear regression models of predictors of plasma ln(DDT) levels [% change in DDT levels (95% CI)] among South African women 20–30 years of age, 2010–2011 by exposure group.^*a*^

Predictor	Unpsrayed (*n *= 175)		non-DDT IRS (*n *= 106)		DDT IRS (*n *= 100)	
	Percent change (95% CI)^*a*^	Adj. *R*^2^	Percent change (95% CI)^*a*^	Adj. *R*^2^	Percent change (95% CI)^*a*^	Adj. *R*^2^
Age (years)
20–22	NS		Referent		NS
23–25	NS		69 (–4, 197)		NS
26–28	NS		270 (79, 665)		NS
29–30	NS		129 (4, 405)	0.03	NS
Education (years)
≤ 11	NS		Referent		NS
12	NS		50 (–5, 137)		NS
> 12	NS		–53 (–77, –7)	0.03	NS
Parity
Nulliparous	NS		Referent		NS
1	NS		–65 (–80, –37)		NS
> 1	NS		–63 (–83, –17)	0.04	NS
Livestock ownership
No	NS		NS		Referent
Yes	NS		NS		95 (26, 201)	0.06
Water source
Public tap	Referent		NS		NS
Piped to yard/home	–73 (–83, –57)	0.16	NS		NS
Butter consumption
< 1 time/month	Referent		Referent		NS
≥ 1 time/month and < 1 time/day	100 (19, 238)		–28 (–57, 22)		NS
≥ 1 time/day	105 (20, 251)	0.02	36 (–21, 134)	0.05	NS
Milk consumption
< 1 time/month	NS		Referent		NS
1–4 times/month	NS		–36 (–61, 5)	0.02	NS
> 4 times/month	NS		–39 (–64, 6)		NS
Egg consumption
< 1 time/month	Referent		NS		NS
1–6 times/month	–29 (–56, 16)		NS		NS
> 6 times/month	–54 (–73, –22)	0.03	NS		NS
Chicken consumption
≤ 1 time/week	NS		Referent		NS
2 times/week	NS		–23 (–57, 39)		NS
3 times/week	NS		–50 (–70, –14)		NS
> 3 times/week	NS		–53 (–73, –17)	0.04	NS
Fish consumption
< 1 time/month	NS		Referent		NS
1–4 times/month	NS		–54 (–72, –24)		NS
> 4 times/month	NS		14 (–35, 100)	0.03	NS
Any pesticide touched open foods
No	NA		NS		Referent
Yes	NA		NS		78 (–1, 221)
Any pesticide touched covering on foods
No	NA		Referent		NS
Yes	NA		84 (13, 200)	0.03	NS
No. of actions taken before IRS
< 4	NA		Referent		Referent
4–6	NA		112 (31, 241)		7 (–37, 82)
> 6	NA		–20 (–52, 31)	0.02	–40 (–63, –0.3)	0.04
Abbreviations: NA, not applicable; NS, not selected*.* All models are adjusted for total lipids. ^***a***^Calculated using the following formula: [exp(β) – 1] × 100.						

Among women in non-DDT IRS households, no single factor accounted for a majority of the explained variance in the fitted models for DDT (Table 3). Rather, a combination of sociodemographic, reproductive, dietary, and IRS-related variables made comparable contributions to the model, with *R*^2^ values for individual predictors ranging from 0.02 to 0.05. Parity was associated with lower DDT levels; women with one live birth had 65% lower DDT levels (95% CI: –80, –37%), and women with more than one live birth had 63% lower DDT levels (95% CI: –83, –17%), compared with nulliparous women. Although women in the older age groups (23–25, 26–28, and 29–30 years) had higher levels of DDT than the youngest age group (69%, 270%, and 129% higher, respectively), the increase was not monotonic. Women who reported that pesticide touched the covering on food after IRS had 84% higher DDT levels (95% CI: 13, 200%) compared with women who reported no pesticide on the covering of food after IRS. Increased chicken and milk consumption were associated with lower levels of DDT. Compared with women who reported eating chicken ≤ 1 time/week or milk < 1 time/month, those who consumed the most chicken (> 3 times/week) had 53% lower DDT levels (95% CI: –73, –17%), and those who consumed the most milk (> 4 times/month) had 39% lower DDT levels (95% CI: –64, 6%). Compared with women who reported fewer than four actions to prepare the home before IRS, women who reported taking four to six actions had higher DDT levels (112%, 95% CI: 31, 241%), whereas little evidence of an effect was observed among women reporting more than six actions (–20%; 95% CI: –52, 31%). The direction of the associations between DDT levels and education (years of schooling) and butter consumption were also inconsistent across the categories of the variables.

Among women in DDT IRS households, those who reported owning livestock had 95% higher DDT levels (95% CI: 26, 201%) than those who did not own livestock (Table 3). Likewise, women who reported that pesticide touched open food after IRS had 78% higher DDT levels (95% CI: –1, 221%) than women who reported that no pesticide touched open food. Compared with women who reported fewer than four actions to prepare the home before IRS, there was little evidence of an effect on DDT levels among women who reported taking four to six actions (7%; 95% CI: –37, 82%). However, women who reported taking more than six actions before IRS had 40% lower DDT levels (95% CI: –63, –0.3%). When this model was run omitting the open foods variable because of potential correlation with the number of actions taken, the estimate for the change in DDT levels associated with taking more than six actions was slightly stronger (–43%, 95% CI: –66, –6%).

Among women in unsprayed villages, water source was the most important predictor of DDE levels (adj. *R*^2^ = 0.09) (Table 4). Women who reported using water piped directly to their yard or home had 61% lower DDE levels (95% CI: –74, –40%) compared with women who reported using a public tap. The only other variable selected in this model was butter consumption; women who consumed the most butter (at least once per day) had 93% higher DDE levels (95% CI: 18, 215%) than women who consumed butter less than once per month.

**Table 4:** Multivariable linear regression models of predictors of plasma ln(DDE) levels [percent change in DDT levels (95% CI)] among South African women 20–30 years of age, 2010–2011, by exposure group.^*a*^

Predictor	Unsprayed (*n *= 175)		non-DDT IRS (*n *= 106)		DDT IRS (*n *= 100)	
	Percent change (95% CI)^*a*^	Adj. *R*^2^	Percent change (95% CI)^*a*^	Adj. *R*^2^	Percent change (95% CI)^*a*^	Adj. *R*^2^
BMI (kg/m^2^)
< 21.6	NS		NS		Referent
21.6–24.7	NS		NS		–49 (–68, –19)
24.8–28.3	NS		NS		–39 (–62, –1)
≥ 28.4	NS		NS		–39 (–63, –1)	0.04
Parity
Nulliparous	NS		Referent		Referent
1	NS		–60 (–76, –32)		–33 (–60, 13)
> 1	NS		–51 (–72, –16)	0.06	–66 (–81, –41)	0.12
Livestock ownership
No	NS		NS		Referent
Yes	NS		NS		87 (31, 167)	0.09
Water source
Public tap	Referent		NS		NS
Piped to yard/home	–61 (–74, –40)	0.09	NS		NS
Butter consumption
< 1 time/month	Referent		NS		NS
≥ 1 time/month and < 1 time/day	85 (13, 202)		NS		NS
≥ 1 time/day	93 (18, 215)	0.03	NS		NS
Milk consumption
< 1 time/month	NS		Referent		NS
1–4 times/month	NS		–16 (–47, 35)		NS
> 4 times/month	NS		–45 (–66, –11)	0.03	NS
Chicken consumption
≤ 1 time/week	NS		Referent		NS
2 times/week	NS		–16 (–52, 47)		NS
3 times/week	NS		–49 (–70, –12)		NS
> 3 times/week	NS		–46 (–69, –6)	0.05	NS
Abbreviations: NA, not applicable; NS, not selected. All models are adjusted for total lipids. ^***a***^Calculated using the following formula: [exp(β) – 1] × 10.						

In non-DDT IRS households, compared with nulliparous women, women with only one live birth had, on average, 60% lower DDE levels (95% CI: –76, –32%) and women with more than one live birth had, on average, 51% lower DDE levels (95% CI: –72, –16%). In addition, among women in non-DDT households, consumption of both milk and chicken was associated with lower DDE levels. Women consuming milk more than four times per month had 45% lower DDE levels (95% CI: –66, –11%) than women who consumed milk less than once per month. Likewise, women who reported eating chicken more than three times per week had 46% lower DDE levels (95% CI: –69, –6%) than women who only ate chicken once per week or less.

In DDT IRS households, parity was the most important (adj. *R*^2^ = 0.12), but not the only, factor associated with DDE levels. Compared with nulliparous women, women with one live birth had 33% lower DDE levels (95% CI: –60, 13%), whereas women with more than one live birth had 66% lower DDE levels (95% CI: –81, –41%). Livestock ownership and BMI were also included in the model of DDE among women in DDT IRS households. On average, women who owned livestock had 87% higher DDE levels (95% CI: 31, 167%) than other women. Higher BMI was associated with lower DDE levels, although compared with parity and livestock ownership, the contribution of BMI to the overall model was small (adj. *R*^2^ = 0.04).

When robust regression analyses were applied to the models, the estimates obtained for both the DDT and DDE analyses remained similar (data not shown). We also assessed the impact of influential data points on our analyses. In the unsprayed village group, 27% (*n* = 47) of observations were influential; among women in non-DDT IRS households, 16% (*n* = 17) of observations were influential, and among women in DDT IRS households, 20% (*n* = 20) of observations were influential. After excluding influential observations, the model selection for DDT and DDE included many of the same predictive variables as the original models (see Supplemental Material, Tables S3 and S4).

## Discussion

The predictors of plasma DDT levels among women in rural South Africa were dependent on whether IRS occurred in the woman’s village or homestead. Several of the predictors identified in the present study may inform targets for interventions aimed at decreasing women’s exposure. Interestingly, water source was the primary predictor of DDT and DDE among women in unsprayed villages. Among women in DDT IRS households, livestock ownership predicted higher plasma DDT and DDE levels, and taking more than six actions to prepare the home before IRS predicted lower plasma DDT levels. Among women in IRS treated villages, regardless of whether the woman lived in a DDT or non-DDT household, parity was the predictor in the DDE models with the highest *R*^2^ value.

The livestock most frequently kept in the villages were chickens, cattle, and, occasionally, goats. Chickens are a primary protein source and, when kept as livestock, have free range throughout the day and are kept indoors at night, providing potential exposure to contaminated air, insects, house dust, and soil (Van Dyk et al. 2010). In a previous investigation among two villages in Limpopo (one IRS village, one not), high levels of DDT and DDE were found in samples of chicken meat, especially in the samples from the IRS village (Van Dyk et al. 2010). In the present study, however, frequent consumption of chicken was not a selected predictor of either DDT or DDE levels among women in DDT IRS households. This variable was included in the final DDT and DDE multivariable models for women in non-DDT IRS households, although this variable was not selected as a predictor after influential observations were excluded from the model. Further, eating chicken was associated with lower DDT and DDE levels, contrary to expectations given the previous findings by van Dyk et al (2010). Although we had crude information regarding the source of foods consumed (participants were asked to judge the proportion of foods eaten that had been personally or locally raised), few of the women (< 10%) reported eating local chickens and it is possible that responding positively to the question regarding livestock ownership was a better indicator of consumption of home-raised meat (including chicken). Although other dietary factors were selected in the final multivariable models among women in unsprayed villages and non-DDT IRS villages, not all were retained after excluding influential observations, and individual dietary factors contributed little to the total explained variance.

Reporting that any pesticide touched open food left in the home during IRS spraying and reporting having taken multiple actions before IRS spraying (such as moving or covering furniture) were also identified as a determinants of DDT levels among women in DDT IRS households. The WHO manual for IRS applicators instructs the applicator to ask the homeowner to remove household items and cover those items that cannot be moved before spraying (WHO 2007). The majority of the women in DDT IRS households reported receiving and complying with directives similar to these [i.e., taking out or covering water and food (most often with a fabric table cloth), moving everything to the middle of the house and covering it with plastic, removing everything from the walls, and going outside the house before spraying occurred]. In addition, women reported receiving and complying with instructions not specifically outlined in the manual (i.e., closing windows and doors, closing cupboards, and packing away or covering clothing). In our study, the reduction in DDT levels associated with homestead preparations was limited to women in DDT IRS households who reported taking more than six actions and the total variance explained by this factor was not large. However, these results highlight one potential opportunity for exposure prevention through education of both residents and IRS applicators.

We found lower levels of DDT and DDE among women in unsprayed villages who had water piped directly to their yard/home compared with women who used a public tap. Interestingly, a previous study of DDT in breast milk also reported a similar finding; mothers relying on piped water had lower mean levels of DDT and DDE in breast milk compared with women who relied on other water sources (Bouwman et al. 2006). Several studies indicate that, even when water contamination exists, levels are often low and unlikely to contribute to residents’ body burden of DDT, particularly among women in unsprayed areas. In one study, of three water samples from unsprayed areas, only one had detectable levels of DDT, and none had detectable levels of DDE (Sereda et al. 2009). In a second study, two of the three water samples from a river in an unsprayed area in Limpopo contained detectable levels of DDE, but not DDT (Barnhoorn et al. 2009). Van Dyk et al. (2010) examined levels of DDT and DDE in various media in two villages in Limpopo (one unsprayed village and one IRS village). Although DDT and DDE were detected in the 12 potable water samples taken from containers in the homes in the IRS village, neither contaminant was detected in any of the 9 water samples from homes in the unsprayed village. In addition, Van Dyk et al. (2010) concluded that water was likely to be contaminated after it is stored on the property because samples taken directly from piped water sources and from the primary river source did not have detectable levels of DDT. This may also explain the present study’s results; that is, women who rely on public sources of water may leave water-filled containers uncovered around their homes, where contamination could occur. However, the water source variable may serve as a surrogate measure of an uncharacterized source of exposure to DDT and DDE. Compared with women who have access to water piped directly to their yard or home, women who rely on public sources of water are more socioeconomically disadvantaged and less educated; they may also rely on local, potentially contaminated, food sources rather than store-bought foods.

The final multivariable model for predictors of DDT levels among women in non-DDT IRS households included a mix of demographic, dietary, and IRS-related variables, and no single variable stood out above the rest in terms of variance explained. Because spray records for individual households were not available, we relied on housing characteristics for discrimination between DDT or pyrethroid use. Although the housing characteristics of this group of women indicated a higher likelihood of IRS with pyrethroids, it is possible that they received IRS with DDT. Also, these women resided in villages alongside neighbors who were likely receiving IRS with DDT, presenting additional exposure opportunities (i.e., pesticide drift due to proximity to DDT-sprayed homes). The findings presented by Ritter et al. (2011) indicate potential DDT exposure through inhalation of indoor and outdoor air, even among populations not directly exposed to DDT through IRS. Individuals within a village may also trade livestock, possibly providing an additional exposure pathway to those whose homes may not be sprayed with DDT if they receive livestock from DDT-sprayed homes. The absence of data regarding the items listed above may have limited the study’s ability to accurately identify determinants of exposure among women in non-DDT IRS households.

The lack of individual spray records for households in IRS villages presents a limitation of the present study. We used housing characteristics to classify women living in IRS villages into two distinct groups based on the likelihood of IRS with DDT, but some misclassification of IRS agent exposure was unavoidable. In addition, our study population may have been augmented with women who were socioeconomically disadvantaged and more motivated by the study reimbursement to participate and thus not entirely representative. Given that the potential determinants of DDT and DDE included in the present study were largely self-reported, it is possible that there is some information bias. Although women were unaware of their own contaminant levels, it is possible, but unlikely, that differential misclassification by DDT or DDE levels occurred if a third factor were both related to contaminant levels as well as the accuracy of women’s report of other influential factors. Given the overall low *R*^2^ values, it is possible that we did not gather information related to potentially important determinants of DDT or DDE. Lastly, although the total number of women in the study was 381, the two IRS groups had only about 100 women each. Studies including a greater number of women within each group would provide more precise effect estimates and would better accommodate statistical analyses of correlated variables, such as the individual variables representing actions taken.

**Figure 1:**
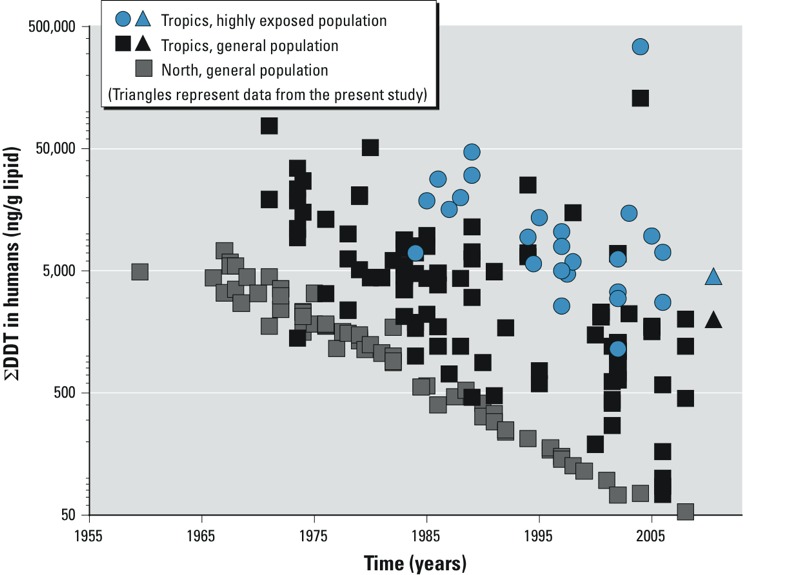
Temporal trends in the sum of DDT levels in human biomonitoring data, adapted from Ritter et al. (2011).

The levels of DDT observed in the present study are similar to levels previously reported. Figure 1 depicts the sum of DDT and DDE levels (nanograms per gram lipids) among women in unsprayed villages and women in DDT IRS households, in relation to data originally presented by Ritter et al. (2011). In Figure 1, the Tropics populations include studies from the following areas: India, Southeast Asia, Africa, and South and Central America. Among these areas, the highly exposed population represents individuals in IRS-treated homes, whereas the general population represents individuals not in IRS-treated homes. The North, general population includes studies among non-Inuits in Greenland, Northern Europe, Canada, and Alaska. The levels of DDT observed in the present study among rural South African women are consistent with what one might expect based on the global trends previously described (Ritter et al. 2011). Although some studies continue to report specific populations with very high levels of DDT (Bouwman et al. 2012), it is reassuring that, overall, levels appear to be decreasing, even among women likely exposed to DDT through IRS. This downward trend may reflect declining use of DDT in agriculture and thereby lower exposures via food and contaminated air.

## Conclusion

Previous studies have advocated for a total homestead environment, or holistic, approach as a context for interpreting and investigating exposures that may occur from a variety of sources in or near the homestead (Bouwman et al. 2011; Sereda et al. 2009; Van Dyk et al. 2010). The present study’s results regarding homestead preparations should be interpreted with caution and regarded as preliminary. Although we report a reduction in DDT (but not DDE) levels among women in DDT IRS households who reported taking more than six actions before IRS, no association was found among women who reported taking four to six actions, and this variable contributed to only a small amount of the total variance. Nonetheless, these results provide evidence that household preparations may serve as an easily modifiable determinant of DDT exposure, and confirmatory studies should follow. These results also provide further support for the total homestead environment approach for the consideration of exposures and exposure reduction strategies, which may include education of residents and spray workers regarding methods to safeguard against DDT exposure in relation to IRS.

## Supplemental Material

(240 KB) PDF
