# Erratum: “Predictors of Plasma DDT and DDE Concentrations among Women Exposed to Indoor Residual Spraying for Malaria Control in the South African Study of Women and Babies (SOWB)”

**DOI:** 10.1289/ehp.122-A180

**Published:** 2014-07-01

The author affiliations were incorrect in the article “Predictors of Plasma DDT and DDE Concentrations among Women Exposed to Indoor Residual Spraying for Malaria Control in the South African Study of Women and Babies (SOWB)” by Whitworth et al. [Environ Health Perspect 122:545–552 (2014); http://dx.doi.org/10.1289/ehp.1307025]. The corrected affiliations appear below and have also been corrected online.

## Kristina W. Whitworth,^1^ Riana M.S. Bornman,^2,3^ Janet I. Archer,^4^ Mwenda O. Kudumu,^4^ Gregory S. Travlos,^5^ Ralph E. Wilson,^5^ and Matthew P. Longnecker^6^

^1^The University of Texas School of Public Health, San Antonio Regional Campus, San Antonio, Texas, USA; ^2^Department of Urology, and ^3^The University of Pretoria Centre for Sustainable Malaria Control, University of Pretoria, Pretoria, South Africa; ^4^Social and Scientific Systems Inc., Durham, North Carolina, USA; ^5^Cellular and Molecular Pathology Branch, and ^6^Epidemiology Branch, National Institute of Environmental Health Sciences, National Institutes of Health, Department of Health and Human Services, Research Triangle Park, North Carolina, USA

*EHP* regrets the error.

