# Supplementary material for: Predictors of Plasma DDT and DDE Concentrations among Women Exposed to Indoor Residual Spraying for Malaria Control in the South African Study of Women and Babies (SOWB)
Source: Environ Health Perspect. 2014 Feb 21;122(6):545–52. doi: 10.1289/ehp.1307025 (PMC4048257; doi:10.1289/ehp.1307025)
Supplement: (240 KB) PDF [file ehp.1307025.s001.pdf]

Environmental Health Perspectives doi:10.1289/ehp.1307025

Supplemental Material

Predictors of Plasma DDT and DDE Concentrations among Women Exposed to Indoor Residual Spraying for Malaria Control in the South African Study of Women and Babies (SOWB)

Kristina W. Whitworth, Riana M.S. Bornman, Janet I. Archer, Mwenda O. Kudumu, Gregory S. Travlos, Ralph E. Wilson, and Matthew P. Longnecker

Table of Contents **Page**

Table S1. Subset of housing variables which best predict plasma DDT levels among South African women in indoor residual spraying villages, based on forward stepwise linear regression model. 2

Table S2. Factor loadings for the single retained factor from the factor analysis to discriminated between DDT and non-DDT sprayed households. 3

Table S3. Multivariable linear regression models of predictors of plasma ln(DDT) levels among South Africa women aged 20-30, 2010-2011, by exposure group, excluding influential observations. 4

Table S4. Multivariable linear regression models of predictors of plasma ln(DDE) levels among South Africa women aged 20-30, 2010-2011, by exposure group, excluding influential observations. 7

Table S1. Subset of housing variables which best predict plasma DDT levels among South African women in indoor residual spraying villages, based on forward stepwise linear regression model.

Variable Name	Adj. R-Squared	AIC Value	p-value
Intercept	0.000	357.10	1.00
Compound	0.061	343.40	< 0.001
Private Water Source	0.080	339.77	0.02
Painted Walls	0.090	338.12	0.06
Dirt Walls	0.093	338.45	0.20

Note: Potential variables also included: plaster walls, brick walls, metal walls, type of toilet, metal roof.

Table S2. Factor loadings for the single retained factor from the factor analysis to discriminated between DDT and non-DDT sprayed households.

Variable Name	Factor Loading
Compound	0.6693
Dirt Walls	0.80776
Painted Walls	-0.49348
Private Water Source	-0.19308

Table S3. Multivariable linear regression models of predictors of plasma ln(DDT) levels among South Africa women aged 20-30, 2010-2011, by exposure group, excluding influential observations (households in unsprayed villages, n = 128; non-DDT IRS households, n = 89; DDT IRS households, n = 80).

Predictor	Unpsrayed: % Change in DDT Levels (95% CI)[a]	Unpsrayed: Adj. R^2	non-DDT IRS: % Change in DDT Levels (95% CI)[a]	non-DDT IRS: Adj. R^2	DDT IRS: % Change in DDT Levels (95% CI)[a]	DDT IRS: Adj. R^2
**Education**						
≤ 11 years	REF		REF		NS	
12 years	-9 (-47, 55)		6 (-41, 91)		NS	
> 12 years	-53 (-75, -11)	0.02	-61 (-84, -7)	0.02	NS	
**Parity**						
Nulliparous	NS		REF		NS	
One	NS		-59 (-80, -18)		NS	
> One	NS		-23 (-63, 61)	0.04	NS	
**Livestock Ownership**						
No	NS		NS		REF	
Yes	NS		NS		65 (4, 161)	0.04
**Water Source**						
Public Tap	REF		NS		NS	
Piped to Yard/Home	-68 (-81, -46)	0.15	NS		NS	
**Ever do Farmwork**						
No	REF		NS		NS	
Yes	-37 (-64, 11)	0.01	NS		NS	

**Predictor**	**Unpsrayed: % Change in DDT Levels (95% CI)[a]**	**Unpsrayed: Adj. R^2**	**non-DDT IRS: % Change in DDT Levels (95% CI)[a]**	**non-DDT IRS: Adj. R^2**	**DDT IRS: % Change in DDT Levels (95% CI)[a]**	**DDT IRS: Adj. R^2**
**Occupational Pesticide Use**						
No	NS		NS		REF	
Yes	NS		NS		-37 (-66, 17)	0.01
**Butter Consumption**						
< 1 time/month	REF		NS		NS	
≥ 1 time/month and < 1 time/day	163 (48, 367)		NS		NS	
≥ 1 time/day	83 (0, 236)	0.04	NS		NS	
**Egg Consumption**						
< 1 time/month	REF		NS		NS	
1-6 times/month	-24 (-56, 33)		NS		NS	
> 6 times/month	-53 (-74, -14)	0.02	NS		NS	
**Fish Consumption**						
< 1 time/month	REF		NS		NS	
1-4 times/month	34 (-19, 120)		NS		NS	
> 4 times/month	301 (33, 1110)	0.01	NS		NS	
**Any Pesticide Touched Open Foods**						
No	NA		NS		REF	
Yes	NA		NS		133 (6, 414)	0.02
**Any Pesticide Touched Covering on Foods**						
No	NA		REF		NS	
Yes	NA		112 (16, 286)	0.03	NS	

Predictor	Unpsrayed: % Change in DDT Levels (95% CI)[a]	Unpsrayed: Adj. R^2	non-DDT IRS: % Change in DDT Levels (95% CI)[a]	non-DDT IRS: Adj. R^2	DDT IRS: % Change in DDT Levels (95% CI)[a]	DDT IRS: Adj. R^2
**Number of Actions Taken Before IRS**						
< 4	NA		REF		NS	
4-6	NA		146 (35, 349)		NS	
> 6	NA		-5 (-49, 78)	0.05	NS	

CI: Confidence Interval; REF: Reference; NS: Not Selected; NA: Not Applicable

Note: All models are adjusted for total lipids.

[a]Calculated using the following forumula: [exp(β)-1]x100.

Table S4. Multivariable linear regression models of predictors of plasma ln(DDE) levels among South Africa women aged 20-30, 2010-2011, by exposure group, excluding influential observations (households in unsprayed villages, n = 128; non-DDT IRS households, n = 89; DDT IRS households, n = 80).

Predictor	Unsprayed: % Change in DDT Levels (95% CI)[a]	Unsprayed: Adj. R^2	non-DDT IRS: % Change in DDT Levels (95% CI)[a]	non-DDT IRS: Adj. R^2	DDT IRS: % Change in DDT Levels (95% CI)[a]	DDT IRS: Adj. R^2
**BMI (kg/m²)**						
< 21.6	NS		NS		REF	
21.6-24.7	NS		NS		-51 (-70, -19)	
24.8-28.3	NS		NS		-41 (-67, 3)	
≥ 28.4	NS		NS		-52 (-73, -16)	0.04
**Parity**						
Nulliparous	NS		REF		REF	
One	NS		-63 (-81, -29)		-34 (-64, 24)	
> One	NS		-51 (-74, -8)	0.06	-73 (-86, -48)	0.13
**Livestock Ownership**						
No	NS		NS		REF	
Yes	NS		NS		83 (22, 173)	0.09
**Water Source**						
Public Tap	REF		NS		NS	
Piped to Yard/Home	-62 (-77, -37)	0.09	NS		NS	
**Occupational Pesticide Use**						
No	NS		NS		REF	
Yes	NS		NS		-37 (-61, -48)	0.02

Predictor	Unsprayed: % Change in DDT Levels (95% CI)[a]	Unsprayed: Adj. R^2	non-DDT IRS: % Change in DDT Levels (95% CI)[a]	non-DDT IRS: Adj. R^2	DDT IRS: % Change in DDT Levels (95% CI)[a]	DDT IRS: Adj. R^2
**Butter Consumption**						
< 1 time/month	REF		NS		NS	
≥ 1 time/month and < 1 time/day	118 (23, 286)		NS		NS	
≥ 1 time/day	74 (-3, 214)	0.03	NS		NS	
**Fish Consumption**						
< 1 time/month	NS		NS		REF	
1-4 times/month	NS		NS		-35 (-58, 0)	
> 4 times/month	NS		NS		-43 (-67, -3)	0.02
**Milk Consumption**						
< 1 time/month	NS		REF		NS	
1-4 times/month	NS		-9 (-47, 55)	0.03	NS	
> 4 times/month	NS		-51 (-72, -13)		NS	
**Meat Consumption**						
< 1 time/month	NS		REF		NS	
1-4 times/month	NS		-32 (-59, 14)		NS	
> 4 times/month	NS		-39 (-70, 21)		NS	
**Any Pesticide Touched Covering on Foods**						
No	NA		REF		NS	
Yes	NA		73 (-1, 201)		NS	

CI: Confidence Interval; REF: Reference; NS: Not Selected; NA: Not Applicable

Note: All models are adjusted for total lipids.

[a]Calculated using the following forumula: [exp(β)-1]x100.
